# Comparative analysis of 14-3-3 isoform expression and epigenetic alterations in colorectal cancer

**DOI:** 10.1186/s12885-015-1856-y

**Published:** 2015-10-30

**Authors:** Gavin M. Young, Vijayababu M. Radhakrishnan, Sara M. Centuori, Cecil J. Gomes, Jesse D. Martinez

**Affiliations:** 1 Undergraduate Biomedical Research Program, University of Arizona Cancer Center, 1515 N. Campbell Ave, Tucson, 85724 Arizona USA; 2 Department of Pediatrics, Steele Children’s Research Center, University of Arizona Cancer Center, 1515 N. Campbell Ave, Tucson, 85724 Arizona USA; 3 Department of Cell & Molecular Medicine, University of Arizona Cancer Center, 1515 N. Campbell Ave, Tucson, 85724 Arizona USA; 4 Cancer Biology Graduate Interdisciplinary Program, University of Arizona Cancer Center, 1515 N. Campbell Ave, Tucson, 85724 Arizona USA; 5 University of Arizona Cancer Center, 1515 N. Campbell Ave, Tucson, 85724 Arizona USA

**Keywords:** qRT-PCR, Colorectal cancer, 14-3-3, DNA methylation, Epigenetics

## Abstract

**Background:**

The 14-3-3 family is a group of intracellular proteins found in all eukaryotic organisms. Humans have seven isoforms that serve as scaffolds to promote interactions of regulatory phospho-proteins involved in many vital cellular processes and previous studies have shown that disturbances in native 14-3-3 levels can contribute significantly to the development of various cancers.

**Methods:**

DNA and RNA was extracted from frozen tissue samples collected by the Human Cooperative Tissue Network. RNA samples were reverse transcribed and subjected to qRT-PCR analysis using fluorescently labelled probes. Genomic DNA was treated with bisulfite and cloned into bacterial vectors for subsequent high-resolution sequencing. Mammalian NIH3T3 cells were transformed with 14-3-3 eta and Ras expression vectors synthesized from cDNA. Colonies were counted and transforming capability assessed after 21 days of growth. Cell lysates were analyzed by western blot to verify protein expression.

**Results:**

Here we examined normal and cancerous 14-3-3 expression levels of all seven isoforms in a cohort of sporadic colorectal adenocarcinomas and in a group of tumors and their matched normals using qRT-PCR analysis. We found a statistically significant decrease in the levels of 14-3-3 sigma, eta, and zeta observed among adenocarcinomas compared to normal tissue. A parallel analysis of microarray data from the TCGA dataset confirmed that expression of sigma and eta were down-regulated in colon tumors. To explore the mechanisms behind 14-3-3 expression changes, we examined the methylation status of the sigma, eta, and zeta gene promoters in selected samples. Our data identified novel CpG methylation sites in the eta promoter consistent with epigenetic silencing of both 14-3-3 sigma and eta isoforms during colon tumorigenesis. Because epigenetic silencing is the hallmark of a tumor suppressor we tested eta in focus formation assays and found that it is capable of suppressing ras-induced transformation of NIH3T3 cells.

**Conclusion:**

To our knowledge, this is the first study to identify the 14-3-3 eta gene as a tumor suppressor and that its expression is suppressed in colon tumors by DNA hypermethylation. These data suggest a link between 14-3-3 expression levels and the development of colon cancers.

**Electronic supplementary material:**

The online version of this article (doi:10.1186/s12885-015-1856-y) contains supplementary material, which is available to authorized users.

## Background

The 14-3-3 proteins are a family of small, highly conserved, acidic proteins with molecular masses of 28–33 kDa. They are found in all eukaryotic species. There are seven different 14-3-3 proteins in mammalian cells, each designated by a Greek letter (β-beta, γ-gamma, δ-delta, ε-epsilon, ζ-zeta, θ/τ-theta/tau, η-eta) and each protein is nearly 80 % similar in their amino acid sequences. There is a highly conserved ligand-binding domain that interacts with phosphorylated serine residues on cellular proteins which targets two high-affinity binding motifs: RSXpSXP (mode1) and RXXXpSXP (mode 2). The ligand-binding domains are the most highly conserved regions of 14-3-3 proteins and all have uniformly high affinity for the binding motifs [1]. It is through these interactions that 14-3-3 proteins exert their biological activity since they have no enzymatic or transcriptional activity of their own.

Although very similar in structure, 14-3-3 proteins serve a number of diverse functions throughout human tissues. Individual regulation of 14-3-3 family members is tightly controlled in many tissues including dermal and epidermal layers [9], bones [14], and developing neurons [2, 17]. Several studies have also shown 14-3-3 proteins play critical roles as signal integration points for cell cycle control, apoptosis, and mitogenic signal transduction [4, 5]. Dysregulation of these proteins has also been linked to several human diseases and that 14-3-3s have even been proposed as potential therapeutic drug targets [31].

14-3-3 proteins have recently come to prominence due to new evidence suggesting they may play a role in human tumorigenesis [29]. For example several studies have shown that 14-3-3 sigma acts as a tumor suppressor and that its expression is often suppressed during the development of breast cancers [12]. Consistent with these findings, 14-3-3 sigma expression is induced by p53 [8] and can suppress the formation of foci induced by ras and myc in rodent cell transformation assays [24]. The expression of other 14-3-3 isoforms, such as gamma and zeta, appears to be upregulated in a number of human tumors [23, 27], suggesting they may exhibit oncogenic properties. Studies in lung and breast cancers have identified a dysregulation of 14-3-3 gene expression in these tumors compared to normal tissue [18, 22]. Loss of 14-3-3 sigma expression remains one of the most consistently observed molecular changes in both breast and colon cancers [3, 20].

To date, only a few studies of human tumors in lung [22], in astrocytomas [30], and in meningiomas [15] have simultaneously characterized the expression levels of all seven 14-3-3 isoforms. The expression levels of the 14-3-3 isoforms have not been characterized in colorectal cancers. Our objective was to determine whether there is a change in expression in relation to normal colonic tissue as well as to identify 14-3-3 genes who’s expression is altered during colon tumorigenesis.

## Methods

### Clinical characteristics of patients and samples

A total of 123 tissue samples were analyzed, taken from 113 patients of varying background: 48 male, 75 female, average age 63 years (range, 15–89 years), 10 black, 99 white, 14 unspecified. Sample set consisted of 71 malignant adenocarcinomas and 52 non-tumor controls including 11 patient-matched pairs. All adenocarcinomas (56 Grade II and 15 Grade III) were confirmed by pathological evaluation and contained a minimum density of 51 % lesion/49 % stroma. Stromal tissue does not appear to contribute significantly to a tissue’s 14-3-3 expression when analyzed by immunohistochemistry [23]. The dataset included patients with varying stages of colorectal cancer (14 Stage I, 28 Stage II, 28 Stage III, and 4 Stage IV). The control group included various diagnoses: 12 diverticulosis, 5 polyps, and 32 non-tumor. Deidentified tissue samples were provided by the Cooperative Human Tissue Network (CHTN Western Division, Vanderbilt University, Nashville, TN) which is funded by the National Cancer Institute. Other investigators may have received specimens from the same subjects. Samples were kept frozen at −70 °C until needed. These studies were reviewed by and designated as exempt by the University of Arizona Human Subjects Protection Program.

### RNA extraction and purification

Total RNA was extracted from tissue samples using the RNeasy® Mini Kit (Qiagen, Hilden, Germany) according to the manufacturer’s guidelines. To preserve RNA integrity, all tissue samples were maintained over dry ice prior to sample disruption using a VDI 12 Tissue Homogenizer (VWR, Radnor, PA). RNA concentration and quality were determined using a NanoDrop® ND-1000 Spectrophotometer (Thermo Fisher Scientific, Waltham, MA).

### Real-time reverse transcription analysis

cDNA was synthesized from 1 μg total RNA using iScript™ cDNA Synthesis Kit (Bio-Rad, Hercules, CA), diluted into 15 μL with nuclease-free water. PCR products were detected with ROX using iTaq™ Supermix (Bio-Rad) and TaqMan® Gene Expression Assays (Applied Biosystems, Foster City, CA) for 14-3-3 gamma (YWHAG, Hs01113553_mH), beta (YWHAB, Hs00268732_m1), epsilon (YWHAE, Hs00356749_g1), zeta (WYHAZ, Hs01122445_g1), theta (YWHAQ, Hs00863277_g1), eta (YWHAH, Hs00607046_m1), and sigma (SFN, Hs00968567_s1). Cycle threshold (C_t_) values for all seven 14-3-3 genes were normalized to GAPDH (Hs02758991_g1, Applied Biosystems).

### DNA extraction and purification

Genomic DNA was extracted from fresh-frozen tissue samples using the QIAamp® DNA Mini Kit (Qiagen), according to manufacturer’s guidelines. Samples were digested with 25 units each of bovine pancreas ribonuclease A (Sigma, St. Louis, MO) for five minutes to remove residual RNA. DNA concentration and quality were determined using a NanoDrop® ND-1000 spectrophotometer (Thermo Fisher Scientific).

### Bisulfite treatment of methylated human DNA

Four matched pairs of tumor and non-tumor genomic DNA (eight samples total) were subjected to bisulfite treatment using EpiTect® Bisulfite Kit (Qiagen), according to the manufacturer’s guidelines. Following column purification, samples were eluted into 10 μL buffer TE (10 mM Tris, pH 8.0; 1 mM EDTA).

### Amplification of 14-3-3 promoter regions from bisulfite-treated DNA

Promoter regions were amplified using primers designed against 14-3-3 sigma (5’- GGTATTGTGAAAGTGGATTTGA -3’ and 5’- ACTATCCAACAAACCCAACAC -3’), 14-3-3 eta (5’- AGTAGGTGAYGTTATTTTGAAA -3’ and 5’- ACCCAACCTCAAAAAATAAC -3’), and 14-3-3 zeta (5’- GGAAATTTTTTTTTTGGTTTGT -3’ and 5’- AATTTTCCTACCCAAATAAAACTTT -3’). When used to amplify bisulfite-treated, genomic DNA, these primer pairs generate products of 702, 651, and 655 base pairs long, respectively. PCR was conducted using Platinum® *Taq* DNA Polymerase (Invitrogen, Grand Island, NY) according to the manufacturer’s guidelines. Reactions were run for 35 cycles using an annealing temperature of 55 °C and 1 μL of template bisulfite-treated DNA. Resultant PCR products were analyzed on a 1 % TAE agarose gel to verify sample quality. DNA bands of the desired mass were excised and subsequently purified using QIAquick® Gel Extraction Kit (Qiagen), according to manufacturer guidelines. Samples were eluted into 30 μL buffer TE for immediate use.

### Single-copy isolation of 14-3-3 promoter regions by subcloning

Purified fragments of 14-3-3 promoter regions were ligated into bacterial cloning vectors using pGEM®-T Easy Vector System I (Promega, Madison, WI) according to the manufacturer’s guidelines. Ligated constructs were immediately used to transform DH5α competent cells and grown on IPTG/β-Gal/Ampicillin positive agar plates for 24 h. Twelve white colonies were randomly selected from each plate and grown in 2 mL liquid cultures overnight. Plasmid DNA was then extracted and purified using AxyPrep Easy-96 Plasmid DNA Kit (Axygen, Union City, CA) according to the manufacturer’s guidelines.

### Sequencing and analysis of bisulfite-treated 14-3-3 promoter DNA

Sequencing of 14-3-3 promoter constructs was conducted by The University of Arizona Genetics Core Facility using an Applied Biosystems 3730 DNA Analyzer. Cloned sequences for each 14-3-3 isoform were aligned against established gene sequences using a ClustalW alignment algorithm and BioEdit^©^ Software (Ibis Biosciences, Carlsbad, CA).

### Plasmids

The T24-C3 vector (activated H-ras inserted into pBR322 plasmid) was obtained from Dr. Radhakrishnan, University of Arizona, AZ, USA. FNpCDNA3 vector was obtained via Addgene (plasmid 45346). The Flag-14-3-3 eta plasmid was created by PCR amplification of 14-3-3 eta cDNA (16) followed by sub-cloning into the FNpCDNA3 vector using BamHI/EcoRI restriction enzymes (NEB, MA).

### Cell culture and transfection conditions

NIH3T3 cell line was obtained from ATCC (American Type Culture Collection). Cells were grown in Dulbecco’s modified minimal essential medium (Cellgro, VA) supplemented with 100 U of penicillin, 100 mg of streptomycin, and 5 % fetal bovine serum (Sigma) and maintained in a humidified atmosphere of 5 % CO_2_.

### Transformation assay

Early passages of NIH3T3 cells were plated in 30 mm, 6-well plates at a density of 300,000 cells/well. 1 μg of each plasmid mixed in FuGene 6 transfection reagent (Promega) was slowly added to culture plates under gentle agitation. After 24 h, the transfected cells were trypsinized and counted. About 5,000 cells were plated into 100 mm dishes, each. Cells were grown in DMEM with 5 % fetal calf serum, changed every 84 h. After 21 days, cells were stained with 0.05 % crystal violet solution (Thermo Scientific #88101) and foci were counted using a ColCount colony counter (Oxford Optronix, UK).

### Western blotting

To verify exogenous protein expression, cell lysates were collected 96 h after transformation. Total protein was extracted and analyzed by SDS-PAGE. Two rabbit-primary antibodies were used to probe for total Ras (Cell Signaling antibody #3339) and Flag peptide (Cell Signaling antibody #2368), incubated overnight in 5 % BSA at a dilution of 1:800. Uniform protein loading was verified using mouse anti-β-actin primary antibody (Sigma #A3853), also incubated overnight in 5 % BSA at a dilution of 1:800. Anti-rabbit HRP-conjugated secondary antibody (Jackson ImmunoResearch antibody #111-035-003) and anti-mouse HRP-conjugated secondary antibody were both used at a 1:10,000 dilution in 5 % blotting-grade blocker (Bio-Rad #170-6404).

### Statistical analysis

Statistics were calculated using R^©^ version 2.13.2 software (R Foundation for Statistical Computing, Vienna, Austria). A two-tailed Student’s *t*-test and Mann–Whitney *U*-test were used to compare differences in 14-3-3 mRNA expression between tumor and non-tumor sample groups. *P*-values less than 0.01 were considered significant.

## Results & discussion

Previous studies have examined changes in the expression of several 14-3-3 isoforms in lung, head and neck, breast, and gastric cancers [10, 13, 22, 27]. Analysis of all seven 14-3-3 family members by immunohistochemistry showed an increase in epsilon, zeta, and theta isoforms in human meningiomas [15] however, there have been no equivalent studies in colon tumors.

**Fig. 1:**
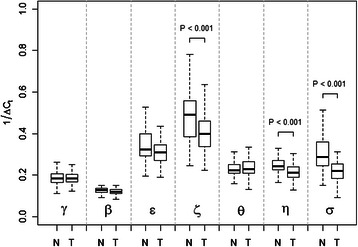
Expression levels of 14-3-3 genes in tumor and non-tumor colon tissues as determined by RT-PCR. Purified whole-RNA extracts taken from frozen tissues were reverse-transcribed and quantified using real-time PCR. Each of the seven human 14-3-3 isoforms are normalized to the housekeeping gene GAPDH (ΔC_t_). Box plot compares expression of all seven 14-3-3 isoforms in tumor (T, *n* = 71) and non-tumor (N, *n* = 52) sample groups. Y-axis is plotted as inverse ΔC_t_. The dark, horizontal bar indicates sample mean and box outlines mark first and third quartiles. Whiskers extend to sample minimum and maximum. Brackets indicate statistically significant differences (determined by Student’s *t*-test, *P* < 0.001)

**Fig. 2:**
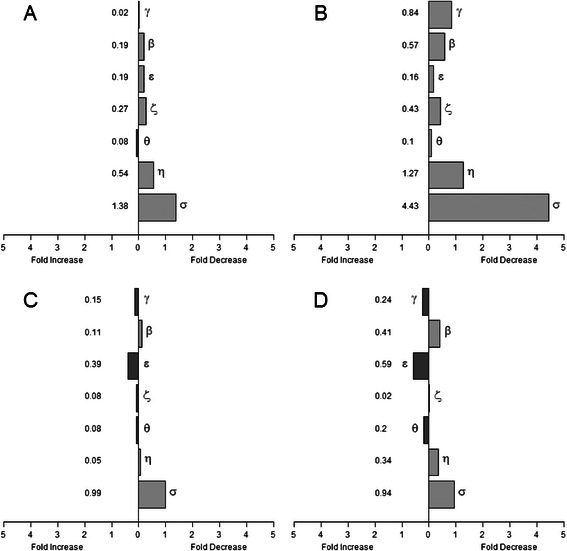
Ratio of 14-3-3 gene expression between tumor and non-tumor sample groups. **a** 14-3-3 expression level comparison of total tumor population with total non-tumor population. Values represent fold difference in the average level of 14-3-3 expression of all 71 tumor samples compared to the average expression level of all 52 non-tumor samples. Fold change = 2^|ΔΔCt|^-1, where ΔΔC_t_ was calculated from the mean of tumor and non-tumor group ΔC_t_ values for each isoform. **b** 14-3-3 expression level comparison of tumor and non-tumor matched pairs. ΔC_t_ values of tumor and non-tumor samples taken from the same patient were compared to each other (ΔΔC_t_) for eleven matched pairs. Values indicated the average fold difference (2^|ΔΔCt|^-1) in expression of each 14-3-3 isoform. **c** 14-3-3 expression level comparison of total tumor population with total non-tumor population of TCGA COAD Dataset. **d** 14-3-3 expression level comparison of tumor and non-tumor matched pairs of TCGA COAD Dataset

In order to characterize 14-3-3 expression in human colorectal adenocarcinomas we began with a cohort of samples that consisted of 71 cancerous and 52 non-cancerous colonic tissues. The relative levels of mRNA expression of each of the 14-3-3 isoforms were determined using primers specific to each isoform in quantitative real-time PCR reactions. The cycle-threshold values (ΔC_t_) were normalized to a GAPDH control. In order to test the quality of our qRT-PCR reactions we also randomly selected the RNA from 24 patients (19 tumor, 5 non-tumor) and performed independent duplicate reactions to test for reproducibility and found that there was less than 1 % variation on average. The expression results were pooled and are graphed in Fig. 1. Statistical analysis revealed that three 14-3-3 isoforms, zeta, eta, and sigma, showed a statistically significant decrease (*p* < 0.001) in mRNA expression in tumor tissue when compared to non-tumor samples whereas little or no change was seen in the other isoforms. In both tumor and non-tumor samples, 14-3-3 isoforms zeta and epsilon exhibited the highest overall expression, consistent with previous 14-3-3 expression data collected in lung tissues [22]. We further analyzed our expression results by calculating the fold differences in mRNA expression in tumor verses non-tumor groups using the mean ΔC_t_ values. The results, graphed in Fig. 2a, reveal that cancerous tissues showed a nearly 1.4 fold drop in expression of 14-3-3 sigma. This is consistent with what has been reported for breast cancers and suggests that depression of 14-3-3 sigma expression may also be important in the development of colorectal tumors. Similarly, the depression of 14-3-3 eta expression suggests that suppressed expression of this isoform is also associated with colorectal tumorigenesis.

For comparison and further evaluation we also analysed a subset of eleven tumors along with their matched normal tissues. Fold differences in expression were calculated for each matched pair individually and averaged (Fig. 2b). We found that 14-3-3 eta and sigma showed a 1.3 and a 4.4 fold decrease in the expression of these genes in tumor compared with their matched normal. Indeed, the magnitude of the fold change in expression of all of the isoforms was increased when tumor samples were compared with matched normals suggesting that using matched normals increases the sensitivity of detection of changes in gene expression. We saw no correlation between expression changes and the patient’s age, sex, or race.

To expand our 14-3-3 mRNA dataset, we analyzed colorectal-adenocarcinoma microarray expression data (normalized, Level 3 data) provided by The Cancer Genome Altas (http://cancergenome.nih.gov/). The dataset was comprised of 155 patients (50 % male, average age at diagnosis: 71, Stage I & II: 48 %, Stage III & IV: 52 %) yielding 155 tumors and 19 non-tumor tissues. Fold changes in mRNA expression of all seven 14-3-3 isoforms were calculated, as before, for all tumors (Fig. 2c) and for matched pairs only (Fig. 2d). TCGA data shows a decrease in 14-3-3 eta and sigma isoforms, supporting our qRT-PCR findings. 14-3-3 depletion becomes more apparent when considering tumor/normal matched pairs again suggesting that using matched pairs can enhance the magnitude of the observed changes (Fig. 2b & d).

**Fig. 3:**
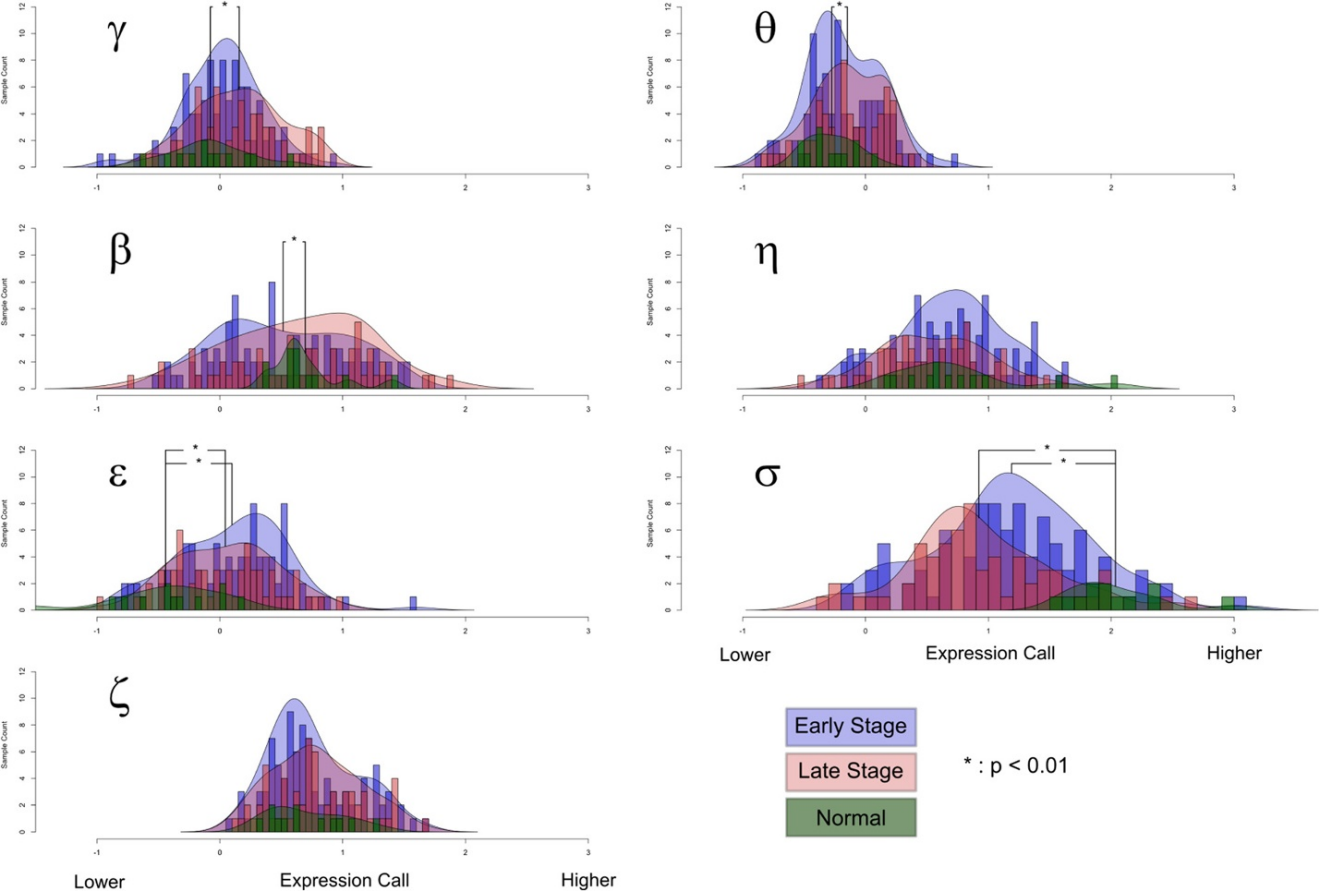
14-3-3 Gene mRNA Expression Data from TCGA. Histograms generated using data from 155 tumors and 19 non-tumor tissues taken from The Cancer Genome Atlas COAD dataset are shown for all seven 14-3-3 genes. Density curves are overlaid highlight distribution. Sample groups are split up into early stage tumors (stage I & II, purple), late stage tumors (stage III & IV, pink), and non-tumor tissues (green). Statistically significant differences in mRNA expression between the populations are shown with brackets

We further analyzed the TCGA microarray data set by examining 14-3-3 gene expression levels and tumor stage. For this analysis we grouped tumor stages I and II together into the early stage group and grouped tumor stages III and IV together into the late stage group. Next we plotted the level of expression for each of the 14-3-3 isoforms for early and late stage tumor groups and compared these to the levels of expression in normal samples. The results are depicted in Fig. 3. As might be expected from previous reports, 14-3-3 sigma showed a statically significant drop in average expression in both early and late stage tumors. Interestingly the magnitude of the level of the decrease in sigma expression became more pronounced in late stage tumors compared to early stage tumors suggesting that repression of this gene increases during tumor development. 14-3-3 eta expression also decreased but did not reach significance and appeared bimodal. That is the drop in expression was more pronounced in some tumors over others. In contrast 14-3-3 epsilon and gamma expression showed a significant increase in both early and late stage tumors. Notably, the average expression level of 14-3-3 beta was reduced in early stage tumors relative to normal cells, but was elevated in late stage tumors. Moreover, change in direction of the expression of this gene (eg. decreased in early stage tumors but increased in late stage tumors) explains why no statistically significant change in expression was observed when the expression results from all tumors was pooled together as was done in Fig. 1.

Having observed decreases in mRNA levels of several isoforms among colorectal tumors, we sought to explore the mechanisms responsible for driving 14-3-3 depletion. Previous studies have indicated that silencing of gene transcription through promoter hypermethylation is a primary mechanism of dysregulation for 14-3-3 sigma in many cancers. Aberrant methylation of the YWHAS gene’s 5’-regulatory region has been reported in cancers of prostate, bladder, and liver tissues [7, 19, 21]. Epigenetic alteration of sigma has shown clinical significance, and hypermethylation correlates strongly with the development of melanomas and squamous cell carcinomas of the vulva [26, 28]. Hypermethylation and down-regulation of 14-3-3 sigma has been consistently observed in a large portion of breast cancers for over a decade and remains a promising biomarker for the disease today [3, 6]. The nature of epigenetic alteration of 14-3-3 and its relationship to cancer, however, appears to be tissue specific. Hypomethylation of 14-3-3 sigma and subsequent up-regulation of the gene has been observed in non-small cell lung carcinomas [25]. The large body of evidence supporting an epigenetic mechanism for regulation of 14-3-3 prompted us to examine the methylation status of the promoter regions of zeta, eta, and sigma.

Although the promoter region and the CpG islands that are methylated in the sigma gene are well documented [16], the promoters and potential methylation sites for zeta and eta are not known. Hence, we began with a computational analysis of all seven of the 14-3-3 promoter sequences using EMBOSS Cpgplot (http://www.ebi.ac.uk/). The predicted methylation sites for all seven of the 14-3-3 genes are shown in supplemental Additional file 1: Figure S1. The predicted CpG islands for eta, zeta, and sigma are displayed in Fig. 3 as dashed lines and represent likely regions of methylation based on GC density. Potential CpG islands predicted to occur in the promoter regions of each of the three 14-3-3 isoforms were used to guide our bisulfite-coupled genomic sequencing efforts. Importantly, the individual CpG methylation sites predicted by EMBOSS and subsequently observed in the bisulfite sequencing of the 14-3-3 sigma gene match previously reported methylation positions [16].

**Fig. 4:**
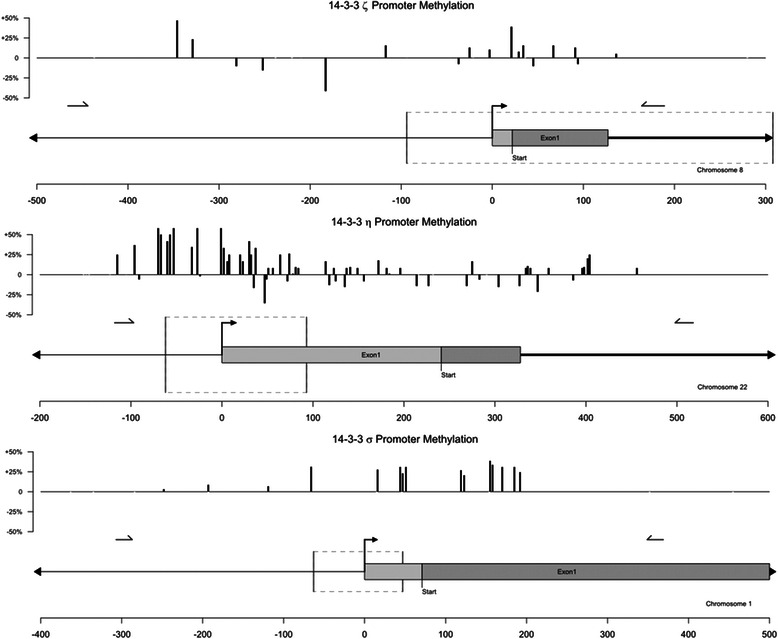
Changes in methylation status of 14-3-3 sigma, eta, and zeta promoter regions. Relative changes in methylation status for each identified CpG site are plotted for all three genes. Upward-facing bars indicate tumor hypermethylation at a particular CpG site, while downward-facing bars indicate hypomethylation. The relative positions of transcriptional start sites and first exons are shown below methylation maps for each isoform. Scales in units of base pairs up or down-stream are shown for reference. Short, horizontal arrows indicate the recognition sites of the forward and reverse primers used to amplify each region. Lightly shaded bars indicate the first exon of each 14-3-3 gene. Start codons for 14-3-3 sigma and eta genes are also shown for reference (coding regions are shown with dark shading). Boxes outlined by dashed lines indicate likely CpG islands (as determined computationally by EMBOSS Cpgplot)

To conduct the analysis genomic DNA was extracted from patient-matched pairs of tumor and non-tumor tissues and subjected to bisulfite treatment to differentiate methyl-cytosines from unmethylated cytosines [11]. PCR amplification of a roughly 650 bp region centered near the start of transcription (depicted in Fig. 4) was conducted for 14-3-3 zeta, eta, and sigma genes. Bacterial subcloning was then used to isolate single copies of the promoter regions for subsequent sequencing and analysis. Figure 4 shows the location of each methylated CpG site that was observed along all three targeted genes. Vertical bars represent average changes in the proportion of methyl-positive clones for matched tumor/non-tumor clones. Positive values indicate an increase in percent methylation of adenocarcinomas compared to their non-tumor controls.

Of the three 14-3-3 genes examined two, sigma and eta, showed significant hypermethylation. The sigma promoter exhibited an overall hypermethylated state (14.0 % methylation of CpG sites in non-tumor tissues versus 34.9 % methylation in tumors), consistent with the epigenetic changes of 14-3-3 established in other cancers [3, 6, 16]. The eta promoter displayed a large number of methylation sites within a roughly 250 base-pair region centered on the gene’s start of transcription. The proportion of methyl-positive CpG sites within 100 bp of the gene’s transcriptional start site was higher in tumor tissues (29.8 %) compared to non-tumor tissues (18.2 %). Such a large shift in promoter methylation suggests that the eta isoform may be dysregulated in a manner similar to 14-3-3 sigma. The zeta isoform did not exhibit strong methylation in either tumor or non-tumor groups, suggesting its observed down-regulation in colon cancers is the result of some unidentified mechanism. To our knowledge, this is the first study that identifies individual CpG methylation sites in the 14-3-3 zeta and eta genes.

**Fig. 5:**
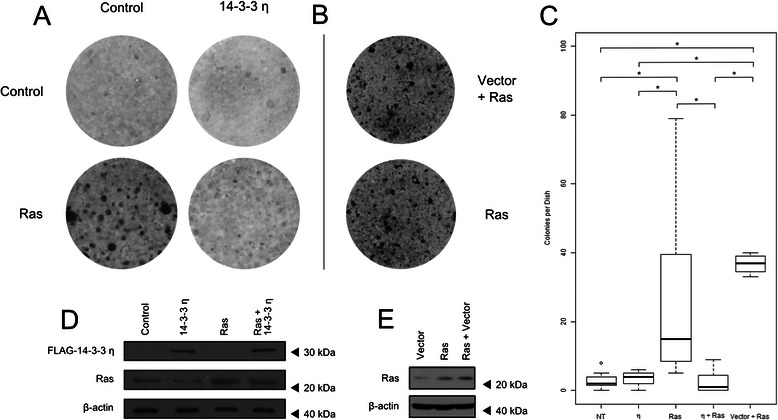
Overexpression of 14-3-3 eta inhibits Ras-induced focus formation in NIH3T3 cells. **a** H-ras and Flag-14-3-3 eta expression plasmids were transfected into NIH3T3 cells as described in the Methods section, replated 24 h after transfection and maintained for an additional 21 days. Subsequently, the cells were stained with crystal violet and the number of foci quantified using an Oxford Optronix colony counter, UK. The experiment was conducted in octuplicate. Representative plates from each of the four experimental groups are depicted. **b** In a parallel control experiment NIH3T3 cells were transfected with H-ras and H-ras plus empty vector and treated as in **a**. The experiment was conducted in octuplicate. **c** The number of foci in panels **a** and **b** were quantified and plotted. Horizontal bars depict the average number of foci per dish. (*: *p* < 0.05, Student’s *t*-Test). **d** A western blot was used to verify the expression of H-ras and N-terminally-tagged Flag-14-3-3 eta proteins in NIH3T3 transformants. **e** A western blot was used to verify expression of H-ras in both Ras and Ras plus Vector group transformants shown in panel **b**

14-3-3 sigma’s established role in colorectal cancers as a tumor suppressor and the similarities in dysregulation of expression of 14-3-3 eta prompted us to test whether eta could also act as a tumor suppressor. A mammalian-expression vector containing full-length 14-3-3 eta was constructed and used to transform human NIH3T3 cell lines. A 900 bp segment of cDNA comprising the entire coding region of 14-3-3 eta was generated (see Methods) and ligated into an FNpCDNA3 vector containing an N-terminal FLAG tag. The multiple cloning site was sequenced to confirm the construct’s integrity and reading frame continuity. The expression plasmid was used in focus formation assays to test whether eta could suppress Ras-induced transformation of NIH3T3 cells. The resultant foci were counted according to the Methods section, results are shown in Fig. 5. H-ras transformants exhibited a statistically-significant increase (*p* < 0.05) in foci count, as compared to non-treated NIH3T3 controls (Fig. 5a & c). This transforming potential of H-ras was significantly suppressed by cotransfection with 14-3-3 eta, a result that is similar to what we observed previously when we cotransfected with 14-3-3 sigma [24]. Cells derived from expanding foci from each transformation assay were grown and collected on day 4 and lysates parepared and analyzed by western blot (see Methods) to confirm exogenous expression of FLAG-14-3-3 eta and H-ras (Fig. 5d). Overexpression of 14-3-3 eta did not increase foci count over the control group, further supporting 14-3-3 eta’s potential role as a tumor suppressor. To verify that the FNpCDNA3 vector alone was not responsible for the tumor suppressor effects seen in the 14-3-3 eta plus H-ras groups, another focus formation assay was run comparing H-ras transformants and H-ras plus empty FNpCDNA3 vector cotransformants (shown in panels b, c, and e of Fig. 5). Ras and Ras plus vector groups showed an equivalent amount of Ras expression by western blot and significantly higher expression than endogenous levels of Ras seen in the vector alone group. Ras and Ras plus empty FNpCDNA3 did not show a statistically significant difference in the number of foci per dish at the end of 21 days.

## Conclusions

Dysregulation of specific 14-3-3 isoforms has been detected in a number of tumor types suggesting that these proteins play a role in maintenance of the normal cell phenotype but can promote tumorigenesis if expression of these genes is altered. Here we examined the expression levels of all seven of the 14-3-3 family members in colorectal adenocarcinomas and found that, in general, the expression of most of the 14-3-3 genes was down regulated in the tumors analyzed by quantitative rtPCR. Of these we found that a statistically significant decrease in 14-3-3 zeta, eta, and sigma expression occurred in tumor samples using rtPCR as compared to non-tumor controls. Comparison of the fold change values obtained for our tumor cohort and the fold change values for the TCGA data set confirm that the expression of sigma and eta are indeed suppressed in colon tumors. However, we found that normalizing using matched controls increased the sensitivity for detecting changes in gene expression. This suggests that the expression level of 14-3-3 genes varies between individuals.

Importantly, analysis of the expression values for colon tumors in the TCGA data set revealed that the change in expression of 14-3-3 genes is complex that we first realized. When compared with tumor stage several 14-3-3 genes exhibited a diverse change in expression. For example, 14-3-3 sigma expression levels, a putative tumor suppressor, decreased in late stage tumors relative to early stage tumors. In contrast the expression of 14-3-3 gamma, an oncogenic 14-3-3 family member, increases in more advanced tumors. However, the most dynamic change in expression was shown by 14-3-3 beta. Here we found that expression of the beta gene was depressed in early stage tumors but increased in late stage tumors. The significance of this change in direction of expression is unclear. However, one possible explanation is that the contribution of this gene to colon tumorigenesis changes with tumor stage. Overall the level and direction of the change in 14-3-3 expression appears to correlate with the degree of advancement of the tumor and appears to be associated with the biological function of the protein.

The expression of some 14-3-3 genes in tumors is thought to be regulated epigenetically. Consequently, prompted by the well documented hypermethylation of the sigma promoter we examined the promoter methylation status of eta and zeta using bisulfite-coupled genomic sequencing and found that the eta promoter was also extensively hypermethylated in colorectal tumors. Indeed the extent of methylation at the eta promoter was considerably more extensive than what we observed for 14-3-3 sigma. Hence, as with 14-3-3 sigma, the expression of 14-3-3 eta is also negatively regulated epigenetically in human tumors suggesting that it may be a tumor suppressor. Consistent with this, 14-3-3 eta could also suppress focus formation induced by an activated Ras oncogene confirming that eta can act as a tumor suppressor.

Overall, our studies show that 14-3-3 gene expression is altered in colon tumors, but that the direction of the change in expression levels varies with each gene and may be a reflection of the role that individual proteins play in the tumorigenic process. Further analysis of how 14-3-3 expression is regulated and how these proteins can influence tumor development is warranted.

## Additional file

Additional file 1: Figure S1. Computer Predicted CpG Islands Near 14-3-3 Promoter Regions. Genomic sequences of all seven 14-3-3 isoforms were analyzed using EMBL’s Cpgplot service. Regions of at least 200 bp in length with a CG density greater than 60 % (using a 100 bp window) are highlighted as red boxes. Height of red boxes indicates the relative density of CG nucleotides within each identified island. Exons for all seven genes are represented by green arrows. (TIFF 357 kb)

## Abbreviations

ATCC: American Type Culture Collection
BSA: bovine serum albumin
CHTN: Cooperative Human Tissue Network
EMBOSS: European Molecular Biology Open Software Suite
GAPDH: glyceraldehyde 3-phosphate dehydrogenase
HRP: horseradish peroxidase
PCR: polymerase chain reaction
qRT-PCR: quantitative real-time polymerase chain reaction
SDS-PAGE: sodium dodecyl sulfate polyacrylamide gel electrophoresis
TCGA: the Cancer Genome Altas
