# Supplementary figures and images for: Comparative analysis of 14-3-3 isoform expression and epigenetic alterations in colorectal cancer
Source: BMC Cancer. 2015 Oct 30;15:826. doi: 10.1186/s12885-015-1856-y (PMC4628284; doi:10.1186/s12885-015-1856-y)

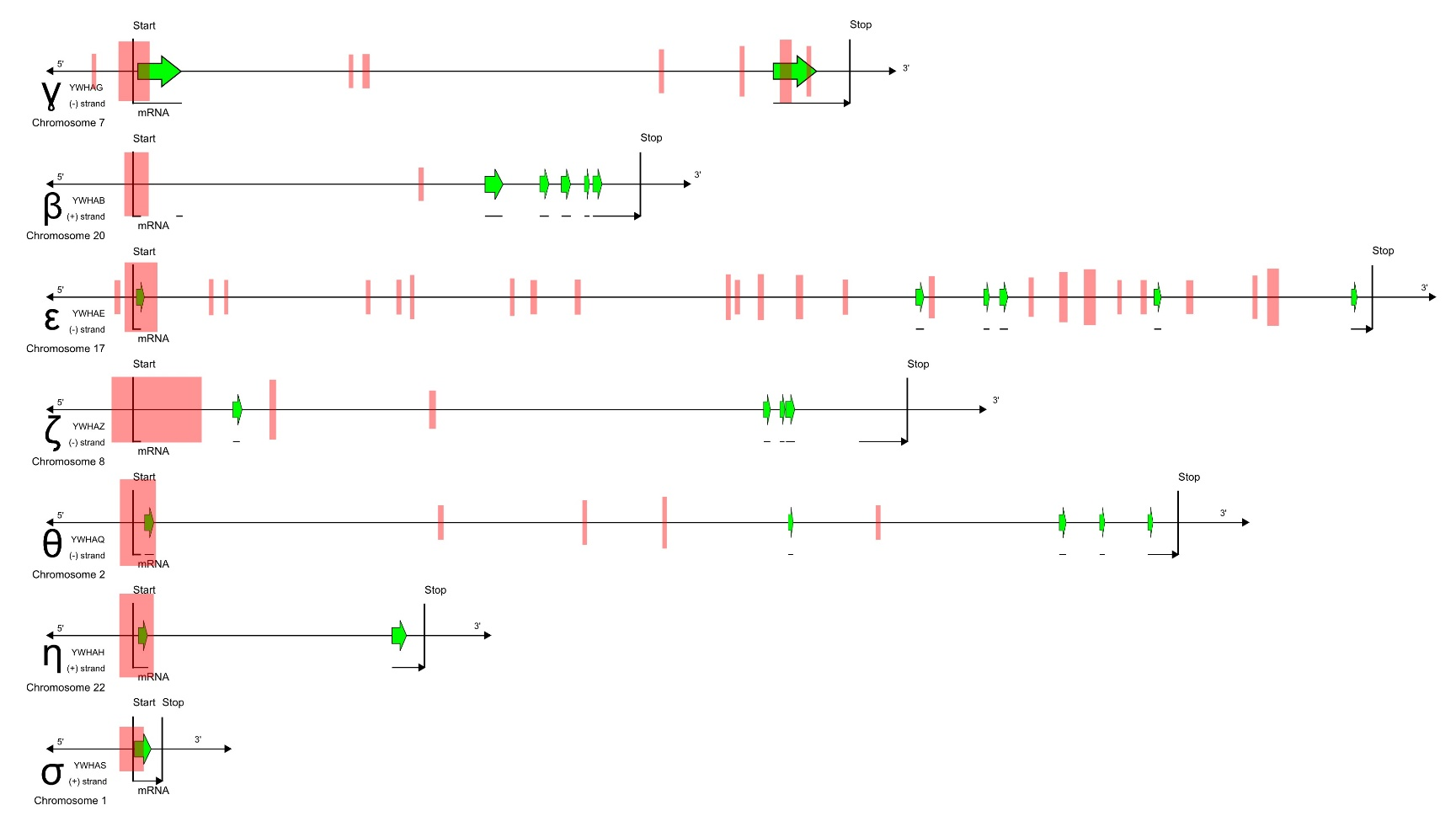

Supplement: Additional file 1: Figure S1. — Computer Predicted CpG Islands Near 14-3-3 Promoter Regions. Genomic sequences of all seven 14-3-3 isoforms were analyzed using EMBL’s Cpgplot service. Regions of at least 200 bp in length with a CG density greater than 60 % (using a 100 bp window) are highlighted as red boxes. Height of red boxes indicates the relative density of CG nucleotides within each identified island. Exons for all seven genes are represented by green arrows. (TIFF 357 kb) [file 12885_2015_1856_MOESM1_ESM.tiff]
